# 2022 Chinese guideline for the management of pregnancy and reproduction in systemic lupus erythematosus

**DOI:** 10.2478/rir-2023-0019

**Published:** 2023-09-27

**Authors:** Xinping Tian, Jiuliang Zhao, Yijun Song, Qian Wang, Mengtao Li, Juntao Liu, Xiaofeng Zeng

**Affiliations:** Department of Rheumatology and Clinical Immunology, Peking Union Medical College Hospital, Chinese Academy of Medical Sciences& Peking Union Medical College; National Clinical Research Center for Dermatologic and Immunologic Diseases, Ministry of Science& Technology; State Key Laboratory of Complex Severe and Rare Diseases, Peking Union Medical College Hospital, Chinese Academy of Medical Sciences & Peking Union Medical College; Key Laboratory of Rheumatology and Clinical Immunology, Ministry of Education, Beijing 100730, China; Department of Obstetrics, Peking Union Medical College Hospital, Chinese Academy of Medical Sciences & Peking Union Medical College, Beijing 100730, China

**Keywords:** systemic lupus erythematosus, reproduction, pregnancy, guideline

## Abstract

Systemic lupus erythematosus (SLE), a prevalent autoimmune disease predominantly affecting women of childbearing age, presents ongoing challenges despite notable advances in diagnosis and treatment. Although survival rates for SLE patients have significantly improved, pregnancy continues to pose a considerable obstacle. Addressing this critical need for enhanced reproductive and prenatal care, there is a pressing imperative to establish standardized protocols for peri-gestational monitoring and treatment in SLE patients. This guideline is jointly sponsored by the National Clinical Research Center for Dermatologic and Immunologic Diseases (NCRC-DID), the Chinese Systemic Lupus Erythematosus Treatment and Research Group (CSTAR), and the Chinese Research Committee of Pregnancy and Reproduction in Autoimmune Rheumatic Diseases (CHOPARD). Thirteen pertinent clinical questions have been generated through several rounds of rigorous clinical and methodological expert discussions and selections for a comprehensive understanding of key aspects in this domain. Guided by thorough examination of research evidence and expert perspectives, the formulated recommendations aim to optimize pregnancy success rates, reduce maternal and infant mortality rates, and ultimately enhance the overall well-being of SLE patients.

Systemic lupus erythematosus (SLE), a systemic autoimmune disease predominantly affecting women of childbearing age, has witnessed significant advancements in clinical management, resulting in improved survival rates among patients. Studies conducted by the Chinese Rheumatism Data Center (CRDC) indicate that the long-term survival rate of SLE patients in China is comparable to international standards.^[1]^ With prolonged life expectancy and enhanced quality of life, individuals with SLE increasingly express desires for marriage and childbirth, raising significant clinical concerns for healthcare providers.^[2,3]^ While the fertility of SLE patients remains comparable to that of non-SLE women of similar age groups, the hormonal fluctuations during pregnancy can trigger or exacerbate SLE flares. Furthermore, numerous factors, including organ damage, autoantibodies, and medications, can significantly impact both the expectant mother and the developing fetus. Extensive global clinical investigations have consistently demonstrated that SLE patients exhibit substantially higher rates of pregnancy-related complications, such as recurrent miscarriage, preterm delivery, fetal death, congenital heart block (CHB), and fetal growth restriction (FGR), compared to non-SLE women.^[4,5]^ Additionally, the maternal mortality rate among SLE patients surpasses that of the non-SLE population by more than 20-fold. Consequently, the imperative to enhance pregnancy success rates while mitigating maternal and infant mortality necessitates an emphasis on strengthened reproductive and prenatal management, alongside the standardization of peri-gestational monitoring and treatment protocols in SLE patients.

The comprehensive management of reproduction and pregnancy in individuals with SLE encompasses a multifaceted process spanning multiple key stages: preconception preparation, SLE monitoring and treatment during pregnancy, fetal monitoring with complications management, postpartum follow-up with breastfeeding, and neonatal monitoring. To address this intricate landscape, professionals from relevant disciplines have collaboratively developed this guideline. The formulation adheres to two essential principles: (1) The primary objective of peri-gestational management in SLE patients is to minimize the intersection between the disease and pregnancy, thereby reducing the incidence of pregnancy complications and optimizing the rates of successful pregnancy, as well as maternal and infant survival. (2) Given the heightened pregnancy risks experienced by SLE patients, adherence to national policies and the consideration of patient and fetal conditions are imperative when applying this guideline to manage SLE patients. To ensure effective implementation, it is strongly recommended that rheumatologists assume leadership roles within multidisciplinary teams comprising specialists from obstetrics, reproductive medicine, family planning, pediatrics, ultrasound imaging, among others. This collaborative approach will facilitate the provision of guidance to patients and their families, enabling the development of optimal plans tailored to individual circumstances.

## Methods

The development of this guideline follows to the rigorous methodologies outlined in the “WHO Handbook for Guideline Development” issued by the World Health Organization in 2014,^[6]^ as well as the “Principles for the Development/Revision of Clinical Guidelines in China (2022 Edition) ” published by the Chinese Medical Association in 2021.^[7]^ Additionally, the guideline compilation incorporates the internationally recognized Reporting Items for Practice Guidelines in Healthcare (RIGHT).^[8]^

1. Guideline sponsors: This guideline has received joint sponsorship from the National Clinical Research Center for Dermatologic and Immunologic Diseases (NCRC-DID), the National Clinical Research Center for Obstetrics and Gynecology, the Chinese Research Committee of Pregnancy and reproduction in autoimmune rheumatic diseases (CHOPARD), the CRDC, and the Chinese Systemic Lupus Erythematosus Treatment and Research Group (CSTAR). The initiation of this guideline took place in March 2021, followed by a thorough review process in March 2022, culminating in the finalization of the guideline in July 2022.

2. Guideline working group: A collaborative and multidisciplinary team has been meticulously assembled for the purpose of developing this guideline. Spearheaded by the Department of Rheumatology and Immunology, the working group encompasses specialists from various fields, including Assisted Reproductive Medicine, Family Planning, Obstetrics, Pediatrics, Cardiac Ultrasound, and Evidence-Based Medicine, among others. Ensuring efficient task allocation, the group is further categorized into four key subcommittees: the Steering Committee, Writing Group, Expert Group, and Evidence Evaluation Group. The Steering Committee, comprising two chief clinical experts and one chief methodologist, assumes the responsibility of overseeing the entire guideline development process, reviewing the guideline’s comprehensive text, and providing expert advice and guidance. The Writing Group consists of domain experts who possess extensive experience in their respective fields. Their primary duties encompass formulating specific clinical questions and refining the recommendations. The Expert Group, composed of experienced professionals, actively engages in voting on the significance of clinical questions and proposing recommendations. Lastly, the Evidence Evaluation Group consists of guideline methodology experts from the Evidence-Based Medicine Center of Lanzhou University/Lanzhou University Grading of Recommendations, Assessment, Development, and Evaluations (GRADE) Center. Their crucial role involves conducting comprehensive evidence retrieval, evaluation, and grading. Importantly, all members of the working group have completed a mandatory Conflict of Interest Disclosure form, thereby affirming the absence of conflicts of interest pertaining to this guideline. This transparency ensures their unrestricted involvement throughout the guideline development process.

3. Guideline registration: This guideline has been registered on the International Practice Guidelines Registration Platform, and the corresponding proposal has been uploaded (Registration number: IPGRP-2022CN298).

4. Guideline users and target population: The intended users of this guideline are physicians specializing in the fields associated with the management of reproduction and pregnancy in individuals with SLE. The guideline primarily targets SLE patients who seek guidance regarding reproduction and pregnancy-related concerns.

5. Selection and determination of clinical questions: To ensure comprehensive and evidence-based coverage, the writing group solicited valuable inputs from a wide range of experts. A synthesis of globally available evidence on clinical manifestations, reproductive protection, preconception consultations, peri-gestational monitoring, treatment strategies, and prognosis pertaining to SLE-related pregnancy and reproduction was conducted, with a particular emphasis on evidence generated within China to represent domestic experiences. Additionally, reference was made to guidelines and consensuses issued by other countries and international organizations regarding the management of pregnancy and reproduction in rheumatic and autoimmune diseases. Following categorization, deduplication, and consolidation, an initial set of 31 clinical questions was formulated based on a comprehensive evaluation of the aforementioned evidence and extensive stakeholder interviews. Subsequently, a Delphi survey was conducted to gauge the perceived importance of each clinical question, utilizing a 7-point Likert scale (ranging from 1 to 7, with higher scores indicating greater importance). New questions were incorporated if they garnered significant attention and recognition by physicians. Finally, a total of 13 key clinical questions were selected for discussion within this guideline, based on a combination of their respective importance rankings and expert opinions.

6. Evidence retrieval: The Evidence Evaluation Group meticulously deconstructed the 13 identified clinical questions into their respective Population, Intervention, Comparison, and Outcome (PICO) components before embarking on an extensive search process. Multiple comprehensive databases were searched, including MEDLINE, Cochrane Library, Web of Science, SinoMed, Wanfang database, and China National Knowledge Infrastructure (CNKI). In addition, official websites of reputable organizations such as the National Institute of Health and Clinical Excellence (NICE), the Scottish Intercollegiate Guidelines Network (SIGN), the American College of Rheumatology (ACR), the European Alliance of Associations for Rheumatology (EULAR), the Asia-Pacific League of Associations for Rheumatology (APLAR), and Google Scholar were also consulted to augment the search breadth. The references in included literature were manually searched for supplementation. This comprehensive search process encompassed all relevant databases from their inception through October 2021. The search strategy was constructed using terms relevant to SLE, pregnancy, neonatal lupus syndrome (NLS), vaccine, *etc*.

7. Inclusion and exclusion criteria of evidence: The main inclusion criteria consisted of (1) The study population was pregnant women diagnosed with SLE or neonates diagnosed with NLS. (2) No restrictions were placed on the intervention, comparison, or outcome measures. (3) The eligible study designs included systematic reviews, meta-analyses, randomized controlled trials (RCTs), cohort studies, case-control studies, and case series studies. Duplications, conference abstracts, and commentaries were excluded. High-quality systematic reviews were directly included to provide support for the recommendations. In the absence of high-quality systematic reviews, recommendations were based on high-quality RCTs. If both systematic reviews and RCTs were lacking, observational studies would be considered.

8. Evidence evaluation and grading: The evidence evaluation group adopted a measurement tool to assess systematic reviews (AMSTAR),^[9]^ Cochrane tool risk of bias (ROB),^[10]^ quality assessment of diagnostic accuracy studies (QUADAS-2),^[11]^ Newcastle-Ottawa Scale (NOS)[^12]^ for the risk of bias assessment of included systematic reviews and meta-analyses, RCTs, diagnostic accuracy studies, and observational studies, respectively. Two investigators independently performed the assessments, and any discrepancies were resolved through discussion or by consulting a third investigator. The Grading of Recommendations, Assessment, Development, and Evaluations (GRADE) approach ^[13]^ was utilized to grade the evidence and formulate recommendations (refer to Table 1).

**Table 1:** Grading of evidence and recommendation

Quality of evidence	Description
High (A)	We have strong confidence in the estimated effect: the true effect is close to the estimated effect.
Moderate (B)	We have moderate confidence in the estimated effect: the true effect is likely to be close to the estimated effect, but it is also possible to be substantially different.
Low (C)	We have limited confidence in the estimated effect: the true effect may be substantially different from the estimated effect.
Very low (D)	We have little to no confidence in the estimated effect: the true effect is likely to be substantially different from the estimated effect.
**Recommendation**	
Strong (1)	Benefits clearly outweigh the risks or the reverse.
Weak (2)	Benefits and risks are closely balanced, or uncertain.

9. Formation of recommendations: The recommendations were formulated by the Expert Group based on the evidence summarized by the evidence evaluation group. The preferences of Chinese patients, as well as the costs and benefits of the interventions, were taken into consideration. A Delphi survey was conducted in March 2022, involving 58 experts, and obtained 78 feedback responses. Through this process, a consensus was reached on all recommendations, defined as an agreement rate of over 85% for each recommendation.

10. External review and approval of recommendations: The recommendations underwent external review by independent reviewers, and revisions were made based on their feedback. Subsequently, the revised recommendations were submitted to the steering committee for final approval.

11. Dissemination and implementation of the guideline: To ensure that physicians and stakeholders comprehensively understand and appropriately apply the recommendations, the guideline working group plans to disseminate and publicize the guideline through various avenues, including (1) introduction in professional journals, websites, and academic conferences, and (2) organizing promotional sessions in some provinces in China.

12. Update of the guideline: A proactive approach to guideline updates is planned, with a timeframe of 3 to 5 years for revising the recommendations. The updates will adhere to international guideline update requirements and guidelines.

## Preconception Preparation of SLE Patients

Recommendation 1.1: We recommend that SLE patients meet the requirements for pregnancy, undergo preconception consultation, and undergo comprehensive risk assessment before planning for pregnancy (strong recommendation, low-quality evidence).

Despite improvements in disease management, SLE patients still face higher maternal and fetal risks compared to healthy women. Therefore, thorough pregnancy planning, preconception risk assessment, and risk stratification are crucial for optimizing pregnancy outcomes.^[14]^ Planned pregnancies in SLE patients have been associated with lower rates of disease flares during pregnancy, with milder flares if they occur. Additionally, there is an increased likelihood of live births and a reduced incidence of adverse pregnancy outcomes. Evidence from the Predictors of Pregnancy Outcome: Biomarkers in Antiphospholipid Antibody Syndrome and Systemic Lupus Erythematosus (PROMISSE) study, a multi-center prospective study conducted in the United States with 385 SLE patients who had inactive or stable active disease, demonstrated that the incidence of adverse pregnancy outcomes was 19.0% (95% CI: 15.2%-23.2%). This included a fetal mortality rate of 4%, neonatal mortality rate of 1%, and preterm delivery rate of 9%. The rates of severe flares during the second and third trimesters were 2.5% and 3.0%, respectively.^[15]^ In a prospective study conducted in China involving 130 SLE patients with planned pregnancies, approximately 29.2% experienced active lupus during pregnancy. Among these cases, 78.9% were classified as mild, 13.2% as moderate, and 7.9% as severe. Adverse pregnancy outcomes were observed in 30.8% of cases, including 28 cases of preterm delivery.^[16]^

Contraception is crucial to prevent unplanned pregnancies in all SLE patients of childbearing age. There are several contraceptive options available for SLE patients, including intrauterine devices (IUDs), barrier contraception, oral contraceptives, and subcutaneous contraceptive implants. Barrier contraception, such as condoms, can be used by all SLE patients and may need to be combined with other contraceptive methods to ensure effectiveness. Prospective studies evaluating the risk of SLE-related thrombosis have shown that combined oral contraceptives containing both progestin and estrogen do not increase the risk of SLE flares in patients with stable SLE. Currently, there is no evidence to suggest that progestin-only contraceptives or IUDs increase the risk of disease flares. However, it is important to note that contraceptives containing estrogen are not recommended for SLE patients who are positive for antiphospholipid antibodies (APLs), with nephrotic syndrome, or with a history of thrombotic events. It is recommended that SLE patients consult with their healthcare provider or specialist to determine the most suitable and safe contraceptive method based on their individual medical history and condition.

Recommendation 1.2: We recommend that SLE patients who have intentions of childbearing undergo preconception consultation and comprehensive assessments. This includes evaluating pregnancy risks, factors associated with adverse pregnancy outcomes, appropriate medication management during pregnancy, and developing a tailored pregnancy plan (strong recommendation, low-quality evidence).

SLE patients with the intention of becoming pregnant should undergo thorough preconception consultation and comprehensive assessments to minimize the risk of adverse pregnancy outcomes (Table 2). The PROMISSE study identified several risk factors associated with maternal and fetal adverse events, which include the presence of lupus anticoagulant (LAC), use of antihypertensive medications, a physician global assessment (PGA) score greater than 1, and thrombocytopenia. In addition to routine assessments conducted for all pregnant women, specific evaluations should be performed for SLE patients, including: (1) SLE activity: Assess SLE activity using the systemic lupus erythematosus pregnancy disease activity index (SLEPDAI)^[17]^ along with PGA. (2) Organ damage: Evaluate for damage in vital organs such as lupus nephritis (LN), hematological abnormalities, cardiac damage, pulmonary hypertension, interstitial lung disease, neuropsychiatric lupus, and other organ damage. Comprehensive preconception assessments for LN should include urine routine tests, urine sediment, 24-hour urine protein, serum creatinine levels, and glomerular filtration rate evaluations. (3) Obstetric history and past thrombotic events. (4) Autoantibodies: anti-cardiolipin antibodies (ACL), anti-β2-glycoprotein I (β2GPI) antibodies, LAC, anti-Sjögren’s-syndrome-related antigen A (anti-SSA) antibodies, and anti-Sjögren’s-syndrome-related antigen B (anti-SSB) antibodies prior to conception. APLs considerably increase the risk of recurrent early miscarriage, intrauterine fetal demise, pre-eclampsia (PE), eclampsia, and hemolysis, elevated liver enzymes, and low platelets (HELLP) syndrome. Anti-SSA and anti-SSB antibodies are associated with cardiac abnormalities and heart block in fetuses. (5) Current medications: Review and adjust current medications based on guidelines for safe medication use during pregnancy in patients with rheumatic and autoimmune diseases. Medications allowed during pregnancy planning may include low-dose glucocorticoids, hydroxychloroquine, azathioprine, and calcineurin inhibitors (cyclosporine A and tacrolimus). The recommended glucocorticoid dose is prednisone ≤15 mg/d or an equivalent dose of non-fluorinated glucocorticoids. However, medications such as methotrexate, leflunomide, mycophenolate mofetil, cyclophosphamide, and thalidomide are contraindicated during pregnancy (see recommendation 4 for further details).

**Table 2:** Contents of preconception consultation and risk assessment in SLE patients

Items	Contents
Demographic information	Age, body mass index, history of smoking and drinking.
Comorbidities	Hypertension, diabetes, hyperlipidemia, and thyroid disease.
Obstetric history	Fetal complications (early miscarriage, fetal death, preterm delivery, fetal growth restriction, congenital heart block, neonatal lupus syndrome, *etc*.). Maternal complications (gestational hypertension, pre-eclampsia, eclampsia, HELLP syndrome, SLE flares, *etc*.).
Past thrombotic events	Arterial and venous thrombosis, heart valve diseases, neurological diseases, *etc*.
SLE activity	Past and current disease activity, recent flares and frequency, contemporary organ damage (especially kidney, heart, lung, and nervous system), *etc*.
Routine examinations	Blood routine test, liver and kidney function test, urine routine test, urine sediment, 24-hour urine protein, electrocardiogram, and echocardiography.
Serological tests	Titer of anti-double-stranded DNA antibodies, level of complement component 3 and complement component 4.
Autoantibodies	Lupus anticoagulant, anti-cardiolipin antibodies, anti-β2-glycoprotein I antibodies, anti-SSA and anti-SSB antibodies.
Current medications	Glucocorticoids: the recommended dose is prednisone ≤15mg/d or an equivalent dose of fluorine-free glucocorticoids. Immunosuppressants: chloroquine, hydroxychloroquine, azathioprine, cyclosporine A, and tacrolimus are allowed, while cyclophosphamide, methotrexate, mycophenolate mofetil, leflunomide, and thalidomide are prohibited when preparing for pregnancy.

SLE, systemic lupus erythematosus.

In addition, it is crucial during the preconception consultation to provide SLE patients and their families with comprehensive and accurate information regarding the risks associated with pregnancy, potential pregnancy-related complications, and the potential adverse outcomes based on individualized risk assessments. Equally important is the understanding of the needs and expectations of SLE patients and their families.

Recommendation 1.3: We suggest that patients with stable SLE consider the use of assisted reproductive technology (ART) if there are indications (weak recommendation, low-quality evidence). We suggest that SLE patients who are positive for APLs receive low-molecular-weight heparin (LMWH) and/or low-dose aspirin during the ART process (weak recommendation, low-quality evidence).

While SLE itself typically does not have a significant impact on fertility, various factors such as advanced age, medication exposure, environmental influences, and some organ damage can potentially affect fertility in SLE patients. A systematic review of 46 studies involving 4704 SLE patients demonstrated that exposure to cyclophosphamide and its cumulative dosage are independent risk factors for premature ovarian failure.^[18]^ As ART continues to advance and become more widely accessible, SLE patients with compromised fertility may consider utilizing these techniques. Existing evidence suggests that the benefits of ART in achieving successful pregnancies outweigh the associated risks for SLE patients with reduced fertility. In vitro fertilization and embryo transfer (IVF-ET) is the most common form of ART utilized. The indications for ART in SLE patients are as follows: (1) Meeting the diagnostic criteria for infertility, which signifies the inability to conceive despite frequent unprotected intercourse for at least one year, and (2) Meeting the indications for IVF-ET, which encompass issues related to gamete transport, endometriosis, ovulation disorders, male factor infertility, immune factors, and unexplained infertility. Performing IVF-ET in SLE patients who meet these indications has shown to be relatively safe and effective. A retrospective study involving 37 women with SLE (*n* = 23, including 8 with positive APLs), SLE with antiphospholipid syndrome (APS; *n* = 4), or primary APS (*n* = 10) described a total of 97 IVF-ET procedures. Among these procedures, eight complications were reported during or after the IVF cycles, including four SLE flares and four thromboembolic events.^[19]^ During the controlled ovarian hyperstimulation procedure of IVF-ET, the significantly elevated serum estradiol levels can potentially trigger SLE flares and increase the risk of arterial and venous thrombosis. Therefore, it is recommended that SLE patients who are positive for APL antibodies but do not meet the Sydney classification criteria for APS [2006] receive a preventive dose of LMWH. For those who meet the classification criteria, a therapeutic dose of LMWH is recommended. LMWH administration should commence during ovarian hyperstimulation, be paused within 24 to 36 h prior to egg retrieval, and resumed after egg retrieval. If the procedure does not result in pregnancy, anticoagulant therapy should continue until the serum estradiol levels return to or near physiological levels.

## Determination of Pregnancy Timing in SLE Patients

Recommendation 2: We recommend that SLE patients contemplating pregnancy fulfill the following criteria: (1) Have stable disease for a minimum of 6 months, (2) Be on oral prednisone at a dose of ≤15 mg/d or an equivalent dose of fluorine-free glucocorticoids, (3) Not be taking potentially teratogenic drugs (including cyclophosphamide, methotrexate, mycophenolate mofetil, leflunomide, Tripterygium wilfordii, *etc*.) for the specified period, (4) Maintain a 24-hour urine protein level ≤0.5 g, and (5) Show no signs of vital organ damage. We do not recommend pregnancy if any of the following conditions are present: pulmonary hypertension, severe restrictive lung disease (*e. g*., forced vital capacity < 1 L), severe heart failure, chronic kidney disease (serum creatinine ≥247 μmol/L), previous intrauterine fetal demise attributed to severe PE/eclampsia/HELLP syndrome, and active disease or stroke in the past 6 months (strong recommendation, very-low-quality evidence).

Due to the absence of high-quality evidence on optimal pregnancy timing and contraindications in SLE patients, this recommendation is primarily based on clinical observational studies and expert consensus.^[20]^ A period of disease remission prior to conception is crucial to minimize the risk of disease flares during pregnancy. Generally, patients meeting the criteria of stable SLE, absence of organ damage, and unchanged medication doses for at least 6 months are considered suitable candidates for pregnancy. However, the optimal duration of disease stabilization remains a topic of debate. The EULAR suggests a duration ranging from 6 to 12 months for pregnancy preparation, taking into account factors such as the extent of organ damage.

The most significant risk factor for adverse pregnancy outcomes in SLE patients is active disease within 6 months prior to conception. The PROMISSE study, which included 384 SLE patients, demonstrated that inactive or stable mild disease at conception is a critical protective factor against SLE flares during pregnancy.^[21]^ Patients with at least 6 months of disease remission before conception had significantly higher rates of term birth (76.47% *vs*. 23.08%) and live birth (80.39% *vs*. 30.77%), along with a reduced incidence of gestational hypertension and PE/eclampsia (9.80% *vs*. 15.38%) compared to those with active disease.^[22]^ Numerous other studies have also shown that active disease preceding pregnancy increases the risks of preterm delivery (Odd Ratio [OR] = 2.75, 95% CI: 1.62–4.92), PE (OR = 4.31, 95% CI: 1.2–15.48), and FGR (OR = 2.48, 95% CI: 1.25–4.92).^[23]^ However, various studies employed different definitions of active disease. Assessments based on SLEPDAI and PGA are recommended with reference to the PROMISSE study. In clinical practice, active disease can be identified by the presence of any of the following conditions: aggravation of organ damage, new-onset organ damage, or the need to escalate the doses of glucocorticoids and/or immunosuppressants to control the disease.

Active LN is an important predictor for adverse pregnancy outcomes. serves as a critical predictor for adverse pregnancy outcomes in SLE patients. Studies have consistently shown that SLE patients with active LN during pregnancy experience significantly higher incidences of lupus flares, fetal loss, PE or eclampsia, preterm birth, and FGR.^[24,25]^ While pulmonary arterial hypertension (PAH) is relatively rare in SLE patients, it is associated with a poor prognosis and high mortality rate during pregnancy.^[26]^ A systematic review analyzed 13 studies including 272 pregnant women with PAH.^[27]^ Among them, 17 had PAH related to connective tissue diseases and the maternal mortality rate was 12%. Causes of death included right heart failure, sudden cardiac death, pulmonary hypertension crisis, PE, and infection. Although advancements in PAH research and the use of targeted drugs have led to improved outcomes in recent years, the mortality rate among pregnant women with PAH still ranges from 5% to 23%.^[28]^ Furthermore, these patients experience a high rate of complications during pregnancy, which warrants the recommendation to avoid pregnancy in individuals with PAH. Next, in SLE patients, moderate or severe renal insufficiency prior to pregnancy can increase the risk of further deterioration of renal function, potentially necessitating renal replacement therapy during pregnancy or postpartum.^[29]^ Additionally, severe interstitial lung diseases in SLE patients can lead to a significant decrease in vital capacity, and a forced vital capacity of less than 1 L is associated with a higher incidence of adverse pregnancy outcomes. Clinical consensus recommends that patients with a forced vital capacity below this threshold should avoid pregnancy or consider therapeutic abortion. ^[30]^ Finally, decompensated heart failure poses a significant risk, increasing the maternal mortality rate. A study on maternal deaths in the United Kingdom revealed that cardiac diseases were the most common cause, accounting for 20% of maternal deaths. ^[31]^

## Follow-Up Schedule for SLE Patients During Pregnancy

Recommendation 3: We recommend that upon confirmation of pregnancy in an SLE patient, a collaborative effort involving rheumatologists and immunologists, obstetricians, and other relevant specialists be undertaken to develop an individualized pregnancy follow-up schedule, closely monitoring the patient’s condition as well as fetal growth and development (strong recommendation, very-low-quality evidence).

Given the complexity and variability of SLE during pregnancy, the involvement of a multidisciplinary team led by rheumatologists has been shown to improve patient management,^[32]^ increase live birth rates, and reduce rates of fetal loss.^[33]^ To effectively mitigate the risks of pregnancy complications, it is essential to establish an individualized pregnancy follow-up schedule based on risk stratification (Table 3).

**Table 3:** Follow-up schedule for SLE patients during pregnancy

Pregnancy weeks	Frequency	Department of rheumatology and immunology	Department of obstetrics
4^th^ week to 10^th^week of gestation	-	Blood routine test, liver and kidney function test, coagulation test, complement test, thyroid function test, antinuclearantibodies, anti-double-stranded DNA antibodies, anti-SSAantibodies, anti-SSB antibodies, antiphospholipid antibodies,urine routine test, urine sediment test, 24-hour urine proteintest, electrocardiogram, and echocardiography.	Screen for HIV, hepatitis B virus, hepatitis C virus,toxoplasma gondii, rubella virus, cytomegalovirus,herpes simplex virus. Perform an ultrasound to confirm pregnancy,determine the expected date of delivery, andmeasure baseline blood pressure and weight.
10^th^ to 16^th^ weeks ofgestation	-	SLEPDAI.	Conduct a nuchal translucency scan and maternalserum screening or non-invasive prenatal testingduring the 11^th^ to 14^th^ week.
16^th^ to 28^th^ weeks ofgestation	Once every 4weeks	SLEPDAI.A fetal echocardiography test every 1 to 2 weeks to monitorthe heart abnormality is recommended for those with anti-SSA/SSB antibodies positive from the 16^th^ week if feasible.	Screen for gestational diabetes during the 24^th^ to28^th^ week. Record fetal heart rate. Conduct fetal echocardiography during the 16^th^to 28^th^ week.
28^th^ to 34^th^ weeks ofgestation	Once every 2weeks	SLEPDAI.	Perform an ultrasound scan every 2 weeks to assess the uterine artery, fetal umbilical artery, fetalductus venosus, and fetal middle cerebral artery.
34^th^ week of gestation to delivery	Once a week	SLEPDAI.	Screen for fetal growth restriction. Determine the types of delivery according to thematernal (*e.g*., hypertension and anticoagulanttherapy) and fetal conditions. Educate on parturient and delivery preparation.
Delivery to 6months afterdelivery	Once every 1-3months	SLEPDAI.Prevention of venous thrombosis.	Breastfeeding education. Postpartum rehabilitation.

SLE, systemic lupus erythematosus; SLEPDAI, systemic lupus erythematosus pregnancy disease activity index.

While there is currently no evidence indicating an optimal monitoring frequency for SLE patients during pregnancy, several studies suggest that follow-up frequency should be determined based on the patient’s specific conditions. It is generally recommended to schedule follow-up visits every 4 weeks prior to the 28^th^ week of gestation, and subsequently increase the frequency to every 2 weeks after the 28^th^ week. However, it is crucial to note that conditions often change rapidly beyond the 28^th^ week, necessitating adjustment of the follow-up frequency in accordance with the patient’s evolving circumstances. Additionally, upon confirming pregnancy, a fetal Doppler ultrasound should be performed to accurately determine the gestational age of the fetus.

In the department of obstetrics, follow-up care includes routine obstetric examinations, regular blood pressure monitoring, and continuous fetal heart rate monitoring. Additionally, starting from the 16^th^ week of gestation, it is recommended to conduct regular fetal Doppler ultrasounds to monitor fetal growth and detect any abnormalities. In cases where FGR or PE is observed, the frequency of follow-ups should be increased accordingly. From the 28th week onwards, Doppler ultrasounds of the fetal umbilical artery should be performed every 2 weeks to assess fetal blood supply, while fetal monitoring should also be conducted every 2 weeks. In the presence of any abnormalities, both the Doppler ultrasounds and fetal monitoring can be performed on a weekly basis.

When anti-SSA antibodies and/or anti-SSB antibodies are positive, it is recommended, whenever feasible, to conduct regular fetal echocardiography tests from the 16^th^ week of gestation to monitor fetal heart structure and conduction (see recommendation 7 for further details).

## Use of Glucocorticoids, Hydroxychloroquine, and Immunosuppressants in SLE Patients During Pregnancy

Recommendation 4: We suggest the use of specific medications, such as oral glucocorticoids, hydroxychloroquine, azathioprine, and calmodulin inhibitors, either alone or in combination, depending on the disease activity and the extent of organ damage, to manage SLE and address any flares or worsening symptoms during pregnancy (weak recommendation, low-quality evidence). We recommend fluorine-free glucocorticoids at the lowest effective dose to control the disease (strong recommendation, low-quality evidence). We suggest the use of hydroxychloroquine throughout pregnancy, unless contraindicated or intolerant (weak recommendation, low-quality evidence). We do not recommend the use of cyclophosphamide, mycophenolate mofetil, methotrexate, or leflunomide during pregnancy (strong recommendation, low-quality evidence).

Medication administration is often necessary for most SLE patients to maintain a stable condition during pregnancy, as this plays a critical role in the well-being of both the patients and their fetuses. However, caution must be exercised during the selection of medications, as some may pose safety concerns before and during pregnancy (Table 4).

**Table 4:** Medication use in SLE patients during pregnancy and lactation period

	Before pregnancy	Pregnancy	Breast feeding
Glucocorticoid	Allowed	No evidence suggests teratogenicity up to now and a minimum effective dose should be maintained.	It is allowed and a minimum effective dose should be maintained. If the dose is >20mg/d, to reduce infant exposure, breastfeeding should be avoided and the milk should be discarded within 4 hours after taking the drug.
Hydroxychloroquine	Allowed	No evidence suggests teratogenicity up to now and it is allowed throughout pregnancy.	The drug can partially enter the milk but do not harm infants.
Azathioprine	Allowed	No evidence suggests teratogenicity up to now and it is allowed throughout pregnancy with a maximum dose of 2mg·kg^-1^·d^-1^.	Allowed
Cyclosporine A	Allowed	No evidence suggests teratogenicity up to now and it is allowed throughout pregnancy with a minimum effective dose.	Allowed
Tacrolimus	Allowed	No evidence suggests teratogenicity up to now and it is allowed throughout pregnancy with a minimum effective dose.	Allowed
Methotrexate	It should be stopped for at least 3 months before pregnancy.	Evidence confirms teratogenicity and it should be prohibited.	Avoided
Cyclophosphamide	Evidence confirms teratogenicity and it should be stopped for at least 6 months before pregnancy.	Evidence confirms teratogenicity and it should be prohibited.	Avoided
Mycophenolate mofetil	Evidence confirms teratogenicity and it should be stopped for at least 3 months before pregnancy.	Evidence confirms teratogenicity and it should be prohibited.	Avoided
Leflunomide	Evidence confirms teratogenicity. Since it can be reabsorbed into the blood through the enterohepatic circulation, cholestyramine is suggested for clearance (8g, 3 times/d, for 11 consecutive days) and then leflunomide need to be stopped for 6 months.	Evidence confirms teratogenicity and it should be prohibited.	Avoided
Sulfasalazine	Allowed	No evidence suggests teratogenicity up to now and it is allowed with a maximum dose of 2g/d. Concurrent folic acid supplementation is required at 5mg/d.	It is allowed with a maximum dose of 2g/d and concurrent folic acid supplementation is required at 5mg/d to avoid folic acid deficiency in infants. It is suggested to be avoided when the infants have confirmed glucose-6-phosphate dehydrogenase deficiency or the infants are premature due to the probability of glucose-6-phosphate dehydrogenase deficiency.
Intravenous immunoglobulin	Allowed	Allowed	Allowed
Rituximab	Allowed	No evidence suggests teratogenicity up to now. It can be used in the first trimester under particular conditions and should be avoided in the second and third trimesters to prevent fetal B cell deficiency.	Avoided
Belimumab	Allowed	No evidence suggests teratogenicity up to now, but it is recommended to be discontinued because of the limited evidence.	Avoided

SLE, systemic lupus erythematosus.

Glucocorticoids serve as the cornerstone of SLE treatment and significantly contribute to favorable maternal and fetal outcomes.^[34, 35, 36]^ Available evidence indicates that glucocorticoids are generally safe for use during pregnancy.^[37,38]^ The placental enzyme 11β-hydroxysteroid dehydrogenase facilitates the degradation of fluorine-free glucocorticoids, thereby prednisone basically does not enter the fetal circulation with a dose < 20 mg/d. However, it is crucial to note that the use of glucocorticoids during pregnancy may increase the risk of hypertension, diabetes, and infection,^[37]^ as well as precipitate fetal growth restriction and premature rupture of membranes.^[39]^ Hence, it is recommended to administer the lowest effective dose of glucocorticoids possible. The recommended maintenance dose is prednisone ≤15 mg/d or an equivalent dose of fluorine-free glucocorticoids. For patients with stable SLE prior to conception, there is typically no need to escalate the glucocorticoid dose during pregnancy. Existing evidence does not support the proactive addition or increment of glucocorticoids as a preventive measure against flares in SLE patients during pregnancy.

Hydroxychloroquine has been shown to effectively reduce disease activity, decrease the risk of flares during pregnancy, improve pregnancy outcomes, prevent PE, and prevent congenital heart block, with no confirmed adverse effects on neonates. ^[40, 41, 42, 43, 44]^ A prospective cohort study conducted in the United States and Canada enrolled 873 pregnant women with autoimmune diseases, of which 279 received hydroxy-chloroquine during pregnancy. The results demonstrated that the use of hydroxychloroquine did not increase the risk of structural birth defects or other adverse outcomes.^[45]^

The use of azathioprine or calmodulin inhibitors, such as cyclosporine A and tacrolimus, during pregnancy has not been associated with fetal abnormalities. In fact, these medications have been linked to lower risks of SLE flares, better disease control, and improved fetal outcomes.^[46, 47, 48, 49]^ Therefore, they may be considered as treatment options if necessary.

However, it is important to note that medications with known teratogenic potential, such as thalidomide, methotrexate,^[50]^ mycophenolate mofetil,^[51]^ cyclophosphamide,^[52]^ and leflunomide,^[53, 54, 55]^ should be discontinued in SLE patients who are planning pregnancy for the required duration of time. Specifically, thalidomide, methotrexate, and mycophenolate mofetil should be stopped for at least 3 months, while cyclophosphamide should be discontinued for 6 months.^[56, 57, 58, 59]^ It is worth mentioning that leflunomide can be reabsorbed through the enterohepatic circulation, and it takes approximately 2 years for the drug to be naturally eliminated. The use of the chelating agent cholestyramine (8 g, 3 times/day) for 11 consecutive days, followed by a 6-month discontinuation of leflunomide, is recommended prior to conception.^[57,60,61]^ A study involving 81 breast cancer patients found that chemotherapy containing cyclophosphamide during the second and third trimesters did not significantly increase the rate of congenital abnormalities.^[62]^ Therefore, in cases where SLE is severely active or life-threatening despite standardized treatment, the use of cyclophosphamide may be considered after thorough communication with patients and their families.

Currently available biologic drugs, such as belimumab, telitacicept, and rituximab, have limited safety data regarding their use during pregnancy. As a result, it is generally recommended to avoid their use unless the potential benefits are deemed to outweigh the risks, and a thorough evaluation of the pros and cons has been conducted with caution.

For male SLE patients who are preparing for pregnancy, the recommended medications include hydroxychloroquine and azathioprine. Additionally, several studies with small sample sizes have indicated that methotrexate,^[56,63]^ mycophenolate mofetil,^[57]^ sulfasalazine, leflunomide, calmodulin inhibitors, and nonsteroidal anti-inflammatory drugs ^[58]^ may be continued if conditions permit. It should be noted that sulfasalazine may have reversible effects on sperm count and quality, but teratogenicity has not been reported. In cases where conception proves difficult, it is advisable to conduct a sperm analysis. Furthermore, cyclophosphamide should be discontinued for at least 12 weeks, and thalidomide should be discontinued for at least 4 weeks prior to male patients preparing for pregnancy. Regarding the use of biologic drugs such as belimumab, telitacicept, and rituximab, data regarding their safety during pregnancy is currently lacking. However, considering that the concentration of IgG in semen is only 10% of that in peripheral blood,^[59]^ and no teratogenic effects have been reported when used in pregnant women, physicians may consider continuing their use after a thorough assessment of the risks and benefits.^[60]^

## Management of Flares or Aggravation in SLE Patients During Pregnancy

Recommendation 5.1: We recommend a stratified treatment approach based on disease severity and organ damage for SLE patients experiencing flares or aggravation during pregnancy (strong recommendation, low-quality evidence). We suggest the use of moderate-dose fluorine-free glucocorticoids for patients with mild disease activity during pregnancy (weak recommendation, very-low-quality evidence). We suggest the use of high-dose fluorine-free glucocorticoids or methylprednisolone pulse therapy for patients with moderate or severe disease activity, and if necessary, these treatments can be combined with immunosuppressants that are deemed safe for use during pregnancy (weak recommendation, low-quality evidence).

Multiple studies have consistently demonstrated a significantly increased risk of flares in SLE patients during pregnancy and up to 3 months postpartum, with rates ranging from 25% to 65%.^[61,64]^ A systematic review encompassing 37 studies and a total of 1842 SLE patients with 2751 pregnancies indicated that pregnancy significantly heightened the risk of flares (flare rate of 25.6%, hazard ratio (HR) = 1.59, 95% CI: 1.27–1.96).^[65]^ SLE flares can occur at any point during pregnancy, with the majority being of mild intensity and presenting as rash, arthritis, mild anemia, mild thrombocytopenia, mild proteinuria, and mildly reduced complement levels. It is noteworthy that the incidence of severe flares during pregnancy is substantially reduced when the disease is stable prior to conception. Furthermore, the use of hydroxychloroquine throughout pregnancy has been shown to decrease the risk of flares.

When SLE flares occur during pregnancy, it is essential to develop an individualized treatment plan that considers disease activity, affected organs, and the extent of organ damage. For patients with mild disease activity, we recommend the use of low-dose fluorine-free glucocorticoids (equivalent to prednisone ≤20 mg/d). These can be combined with immunosuppressants to minimize the cumulative glucocorticoid dose throughout pregnancy, reducing the risk of long-term adverse reactions. In cases of moderate to severe disease activity, we recommend the use of fluorine-free glucocorticoids at a dose equivalent to 1 mg·kg-1·d-1 of prednisone or methylprednisolone pulse therapy combined with immunosuppressants. Pulse therapy allows for rapid disease control without significantly increasing the incidence of adverse events. The dosage of glucocorticoids and immunosuppressants should be adjusted based on disease activity and the gestational week. It is important to note that glucocorticoids with a dose equivalent to > 10 mg/d of prednisone can increase the risk of preterm delivery, premature rupture of membranes, and fetal growth restriction ^[66]^ Therefore, it is crucial to minimize glucocorticoid dosage as soon as the disease is under control. Intravenous immunoglobulin (IVIG) therapy is another viable treatment option during pregnancy, as it can partially alleviate hematological and renal damage.

Recommendation 5.2: We recommend that LN flares in SLE patients during pregnancy should be differentiated from gestational hypertension, PE, and eclampsia (strong recommendation, very-low-quality evidence). We suggest the use of moderate-dose fluorine-free glucocorticoids for patients with mild LN (weak recommendation, very-low-quality evidence). We suggest the use of high-dose fluorine-free glucocorticoids or methylprednisolone pulse therapy for patients with moderate to severe LN, and if necessary, these treatments can be combined with immunosuppressants that are deemed safe for use during pregnancy (weak recommendation, low-quality evidence).

LN represents one of the most prevalent forms of organ damage in SLE patients, and its incidence significantly increases during pregnancy. Moreover, patients diagnosed with LN prior to conception face a higher risk of LN flares during pregnancy (OR = 2.66, 95% CI:1.51–4.70). In a study analyzing 113 pregnancies in 81 SLE patients with LN, the incidence of LN flares during and after pregnancy was approximately 30%.^[67]^ Another study found that 75% of SLE patients who experienced flares during pregnancy had LN. Mild proteinuria can occur in healthy pregnant women due to increased renal blood flow. SLE patients may exhibit elevated urinary protein levels during pregnancy due to various underlying causes, requiring careful differentiation. Both PE/eclampsia and LN flares can manifest as increased urinary protein excretion, lower extremity edema, hypertension, and renal function impairment. Consequently, distinguishing between the two conditions can be challenging, and definitive conclusions may only be reached after pregnancy termination or based on responses to empirical treatment. Reduced levels of complement C3, elevated anti-double-stranded DNA antibody titers, active urine sediment, and other SLE flare manifestations such as rash, arthritis, and hematological abnormalities may support the diagnosis of LN flares. It is crucial to be aware that LN flares and PE can coexist.

Patients experiencing mild LN flares can be treated with fluorine-free glucocorticoids at a dose equivalent to < 0.5 mg·kg^-1^·d^-1^ of prednisone. For moderate to severe flares, oral or intravenous infusion of high-dose glucocorticoids or methylprednisolone pulse therapy, along with IVIG, can be considered. Additionally, β-blockers and calcium-channel blockers can be utilized to manage blood pressure. It is important to note that angiotensin-converting enzyme inhibitors (ACEIs) and angiotensin II receptor blockers (ARBs) are contraindicated during pregnancy due to the potential risks of irreversible fetal renal impairment, fetal loss, and teratogenicity.^[68]^ As the pregnancy approaches full term, close monitoring of blood pressure and PE-related symptoms becomes crucial in addition to LN treatment. If necessary, delivery is recommended to alleviate potential symptoms of PE, and prompt initiation of LN treatment should be implemented.

Recommendation 5.3: We suggest that if thrombocytopenia occurs in SLE patients during pregnancy, treatment should be given after a comprehensive analysis of the severity of thrombocytopenia, related clinical manifestations, gestational age, SLE activity, and previous medication usage. Options to consider include fluorine-free glucocorticoids, IVIG, calmodulin inhibitors, and azathioprine (weak recommendation, low-quality evidence).

Thrombocytopenia represents a common clinical manifestation of SLE flares during pregnancy. A retrospective study conducted in China reported that the incidence of thrombocytopenia in SLE patients during pregnancy ranged from 23% to 54%, occurring at any stage of pregnancy, including the postpartum period. The etiology of thrombocytopenia in SLE patients during pregnancy is multifactorial and complex, encompassing SLE flares, thrombotic microangiopathy, hypertensive disorders in pregnancy (such as PE/eclampsia and HELLP syndrome), infections, and medications. To differentiate pseudothrombocytopenia caused by platelet aggregation from thrombotic microangiopathy characterized by red blood cell fragmentation, a blood smear examination can be performed. In cases where necessary, a bone marrow cell morphology examination can be conducted to identify underlying bone marrow disorders. Evidences from CSTAR, a prospective multicenter registry study conducted in China, demonstrated that leukopenia, LN, hypocomplementemia, and elevated SLE disease activity index (SLEDAI) scores were independent risk factors for SLE-associated thrombocytopenia.^[69]^ Additionally, a systematic review revealed a significantly higher risk of thrombocytopenia in SLE patients with positive APLs (OR = 2.48, 95% CI:2.10–2.93).^[70]^ Furthermore, in pregnancies complicated by PE or HELLP syndrome, the incidence of thrombocytopenia ranges from 5% to 21%.

The management of thrombocytopenia in patients with SLE requires careful differentiation of the underlying etiologies. It is important to note that the treatment goal is to maintain a platelet count sufficient to prevent bleeding rather than to normalize the count. Currently, there is a lack of high-quality evidence or recognized expert consensus/guidelines regarding the treatment of thrombocytopenia in SLE patients. Present clinical practice mainly refers to guidelines for the management of immune thrombocytopenia.^[71,72]^ Glucocorticoids and IVIG can be used as first-line therapy. Specifically, IVIG is recommended at a dose of 400 mg·kg^-1^·d^-1^ for 3 to 5 days, with the option of repeating the treatment after 1 to 2 weeks if necessary. In cases of severe thrombocytopenia with a serious and life-threatening bleeding risk, consideration can be given to intravenous methylprednisolone pulse therapy. If patients are dependent on or resistant to glucocorticoids, recombinant human thrombopoietin alone or in combination with approved immunosuppressants during pregnancy, such as calmodulin inhibitors (cyclosporine A and tacrolimus) and azathioprine, can be considered as potential therapies. When the platelet count exceeds 50×109/L, vaginal delivery is generally considered safe. For patients requiring cesarean section under epidural anesthesia, platelet transfusion should be considered if the platelet count remains below the level deemed safe for surgery despite standardized treatment.

Recommendation 5.4: We suggest considering termination of pregnancy before the 22^nd^ week of gestation in cases where PAH is new-onset or worsens during pregnancy (weak recommendation, very-low-quality evidence). We suggest that if the pregnancy continues, patients should be referred to specialized PAH centers for further evaluation, treatment, and follow-up. Potential therapies such as prostanoids and/or phosphodiesterase-5 inhibitors can be considered (weak recommendation, very-low-quality evidence).

Some SLE patients may develop new-onset PAH during pregnancy, although the underlying mechanisms remain unclear. The elevated levels of progesterone and estrogen during pregnancy can lead to increased pulmonary blood flow, resulting in right heart overload. The third trimester further exacerbates the overall cardiac burden. Additionally, during delivery, the rise in intra-abdominal pressure and the associated stress response cause significant hemodynamic changes, with each contraction resulting in 300 to 500 mL of blood returning to the systemic circulation from the placenta. Following fetal delivery, the sudden decrease in intra-abdominal pressure significantly enhances stroke volume and cardiac output, posing a relatively high risk of cardiogenic shock during the prenatal period.^[73]^ In light of this, it is recommended to consider terminating the pregnancy before the 22^nd^ week of gestation for patients experiencing new-onset PAH during pregnancy. Those who choose to continue the pregnancy must be promptly referred to specialized PAH centers with expertise in the condition for comprehensive evaluation and close monitoring. Targeted drugs for PAH, such as phosphodiesterase-5 inhibitors (sildenafil and tadalafil) and prostanoids (iloprost and treprostinil), are considered acceptable treatment options during pregnancy. However, endothelin receptor antagonists and soluble guanylate cyclase stimulators are contraindicated due to confirmed teratogenic effects.

## Management of SLE Patients with Positive APLs During Pregnancy

Recommendation 6: We recommend individualizing treatment for SLE patients with positive APLs during pregnancy based on risk stratification, taking into account their prior history of pathological pregnancy, prior history of thrombosis, and specific types of positive APLs. Low-dose aspirin and/or LMWH are recommended (strong recommendation, high-quality evidence). We recommend the use of hydroxychloroquine throughout pregnancy, if not contraindicated or intolerant (strong recommendation, moderate-quality evidence).

The APLs profiles primarily encompass ACL, anti-β2GPI antibodies, and LAC,^[74]^ with approximately 30% of SLE patients testing positive for APLs. Some patients exhibit persistent medium-to-high titers of APLs along with a history of pregnancy morbidity, including recurrent miscarriage, pregnancy loss after the 10th week of gestation, and PE/eclampsia, which are collectively referred to as obstetric APS. Numerous studies have demonstrated that individuals with persistent positive APLs have a higher occurrence of various pregnancy complications. However, appropriate management and standardized treatment can significantly improve pregnancy outcomes. According to the Sydney classification criteria [2006] for APS, established by the International Society on Thrombosis and Hemostasis, pregnancy morbidities associated with APLs can be categorized into three types: (1) At least one unexplained fetal death beyond the 10^th^ week of gestation, with confirmation of normal fetal morphology through ultrasound or direct examination; (2) At least one preterm delivery of morphologically normal neonates before the 34^th^ week of gestation due to eclampsia, severe PE, or severe placental insufficiency; (3) At least three consecutive unexplained spontaneous abortions before the 10^th^ week of gestation, with exclusion of other causes such as maternal anatomical and hormonal abnormalities, and fetal, maternal, and paternal chromosomal abnormalities. In clinical practice, some patients fulfill the laboratory criteria of the Sydney classification for APS but not the clinical criteria. These patients may experience two consecutive unexplained abortions, at least three non-consecutive unexplained abortions, late-onset PE, placental abruption, or late preterm birth. They are classified as having non-criteria obstetric APS, which entails a risk of recurrent pathological pregnancy. However, with standardized treatment for obstetric APS (*i.e*., aspirin combined with LMWH), these patients show improved pregnancy outcomes.^[75]^

The efficacy of aspirin and/or heparin in improving pregnancy outcomes among APLs-positive patients has been confirmed by multiple studies.^[76,77]^ A systematic review, which included 11 studies (9 RCTs and 2 quasi-RCTs) with a total of 1672 female patients with persistent positive APLs, demonstrated that the combination of aspirin and heparin (either unfractionated heparin or LMWH) significantly increased the live birth rate (risk ratio (RR) = 1.27, 95% CI:1.09–1.49) compared to aspirin alone, without serious adverse events or congenital malformations.^[78]^ Based on several international guidelines, treatment stratification should be conducted considering obstetric clinical manifestations, prior history of thrombosis, and the specific types of positive APLs. This approach guides the development of an individualized treatment strategy for APLs-positive patients as follows: (1) SLE patients with positive APLs, recurrent early miscarriage or late pregnancy loss, and no prior history of thrombosis. It is recommended to initiate low-dose aspirin therapy (50–100 mg/day) when attempting to conceive and to start prophylactic doses of LMWH once the intrauterine pregnancy is confirmed. A study with a small sample size indicated that the combination of aspirin and heparin significantly reduced the rate of pregnancy loss and increased the rate of live births compared to aspirin alone among APS patients.^[79]^ (2) APS patients with a history of preterm delivery related to placental insufficiency but no prior thrombosis: It is recommended to initiate aspirin therapy at a dosage of 50–100 mg per day starting from the first trimester and continue throughout the pregnancy. In the meantime, prophylactic doses of LMWH can be administered. If the use of aspirin alone proves ineffective, further examination of the placenta may reveal significant decidual cell inflammation, vascular lesions, or the formation of thrombosis. In such cases, a combination of aspirin and a therapeutic dose of LMWH should be applied during pregnancy. (3) APS patients with a prior history of thrombosis: A therapeutic dose of LMWH should be given during pregnancy for anticoagulation. If these patients also have a history of pathological pregnancies, it is recommended to supplement LMWH therapy with aspirin during pregnancy. (4) SLE patients with APLs positive but no related clinical manifestations: High-quality systematic reviews and clinical evidence lack. In such cases, the recommended treatment is aspirin alone at a dosage of 50–100 mg per day. (5) Patients with refractory obstetric APS: This is defined as experiencing adverse pregnancy outcomes despite standardized treatment with aspirin and LMWH. There is currently no second-line treatment supported by high-quality systematic reviews or clinical studies. However, a recommended approach is to begin treatment before pregnancy with a combination of aspirin and hydroxychloroquine. Additionally, starting from the first three months of pregnancy, low-dose prednisone (≤10 mg per day) or an equivalent dosage of fluorine-free glucocorticoids should be initiated. Limited data suggest that IVIG and therapeutic plasma exchange may be effective, but further confirmation is needed through large-scale and well-designed clinical studies. It has been proven that high-dose glucocorticoids and cytotoxic drugs such as cyclosporine A are ineffective and may lead to adverse events in some patients.

## Prevention, Identification, and Treatment of Fetal Heart Block

Recommendation 7: We suggest conducting echocardiographic surveillance every two weeks, starting from the 16^th^ week until the 26^th^-28^th^ week of gestation, in order to detect fetal heart block as early as possible in SLE patients who test positive for anti-SSA and/or anti-SSB antibodies (weak recommendation, low-quality evidence). We recommend initiating hydroxychloroquine before conception, unless contraindicated, to prevent fetal cardiac abnormalities (strong recommendation, low-quality evidence). We suggest he administration of oral dexamethasone in cases where the fetus develops arrhythmias, heart valve diseases, cardiomyopathy, or endocardial fibroelastosis (weak recommendation, low-quality evidence).

Starting from the 16^th^ week of gestation, anti-SSA and/or anti-SSB antibodies can cross the placental barrier through active transport, causing antibody-mediated damage to the fetal heart. This can affect various components of the fetal heart, including the atrioventricular conduction system, myocardium, endocardium, and heart valves, resulting in manifestations such as arrhythmias, dilated cardiomyopathy, endocardial fibroelastosis, and heart valve diseases.^[80,81]^ Early identification and diagnosis of fetal heart block can be facilitated through the serial fetal echocardiographic surveillance. While a small observational study suggests that hydroxychloroquine may partially prevent CHB in connective tissue disease patients, conflicting evidence exists regarding effective treatment. Therefore, treatment strategies should be carefully formulated based on individual patient conditions, considering the potential benefits and risks.

Anti-SSA and/or anti-SSB antibodies-associated fetal heart block is not exclusive to pregnant patients with SLE and Sjogren’s syndrome but can also occur in asymptomatic carriers of these autoantibodies. Generally, the incidence of fetal heart block in the initial pregnancy ranges from 1% to 2% among individuals positive for anti-SSA and/or anti-SSB antibodies, with a higher likelihood in those with hypothyroidism. Additionally, the incidence significantly increases to 17%-19% in subsequent pregnancies for those with a previous history of fetal heart block. The primary manifestations of fetal heart block associated with these antibodies include arrhythmias, dilated cardiomyopathy, endocardial fibroelastosis, and heart valve diseases, with atrioventricular block (AVB) being the most common. Third-degree AVB often indicates a poor prognosis, with intrauterine fetal demise occurring at a rate of 10% to 29%, and a postnatal permanent pacemaker implantation rate ranging from 63% to 93%.^[82, 83, 84]^ A ventricular rate of less than 55 beats per minute serves as an important risk factor for severe adverse fetal outcomes. Other risk factors include hydrops fetalis, increased cardiothoracic ratio, aortic valve regurgitation, and decreased aortic flow velocity. While some fetuses with first- and second-degree AVB can be cured, others can progress to third-degree AVB. Various types of arrhythmias can also be observed, such as sinus bradycardia, atrial bradycardia, QT prolongation (≥440 ms), and pre-excitation syndrome. Endocardial fibroelastosis may occur alone or in combination with AVB. Fetal cases with mild endocardial fibroelastosis generally have a favorable prognosis, characterized by enhanced echoes in the region of the chordae tendineae, heart valves, and atrial wall in echocardiography. Conversely, those with severe endocardial fibroelastosis, complicated by arrhythmias or dilated cardiomyopathy, often experience poor clinical outcomes. Late-onset dilated cardiomyopathy occurs in approximately 10% of children with AVB and can also manifest independently.^[85,86]]^

For all pregnant women with anti-SSA and/or anti-SSB antibodies, it is important to consider their fetuses at high risk for cardiac abnormalities. Proactive screening, early identification, and timely intervention are now widely agreed upon by physicians. Currently, the most commonly used and effective screening method is fetal echocardiography. It is recommended that pregnant women positive for anti-SSA and/or anti-SSB antibodies undergo a fetal echocardiography every two weeks, starting from the 16^th^ week until the 26^th^-28^th^ week of gestation.

During the fetal echocardiography, it is advised to employ spectral Doppler to measure the atrioventricular time interval. A value of ≥140 ms indicates the possibility of cardiac conduction abnormalities, requiring closer monitoring through weekly fetal echocardiography or intervention. Fetuses with an atrioventricular time interval ≥150 ms can be diagnosed with first-degree AVB, and active treatment should be initiated to prevent progression to third-degree AVB. Furthermore, the fetal echocardiography allows for the assessment of heart chamber sizes, ventricular function, and valvular function.

The pre-conception initiation of hydroxychloroquine has shown promising results in reducing the incidence and recurrence rate of fetal heart block associated with anti-SSA and/or anti-SSB antibodies. A multicenter retrospective cohort study analyzed 257 pregnant women who tested positive for anti-SSA and/or anti-SSB antibodies and had a history of neonates with cardiac manifestations of NLS, such as AVB. Among the subjects, 40 received hydroxychloroquine while 217 did not. The incidence of NLS cardiac manifestations (including second-degree and third-degree AVB and cardiomyopathy) was 7.5% in the hydroxychloroquine group compared to 21.1% in the non-hydroxychloroquine group. Notably, there were no fetal deaths in the hydroxychloroquine group, whereas the case fatality rate for fetuses with cardiac manifestations in the non-hydroxychloroquine group was 22%. Multivariate analysis demonstrated that hydroxychloroquine significantly reduced the recurrence rate of NLS cardiac manifestations (OR = 0.23, 95% CI:0.06–0.92).^[87]^ Furthermore, in a multi-center, open-label, single-arm clinical trial, 54 patients with a history of fetal heart block initiated a daily dose of 400 mg of hydroxychloroquine before the 10th week of subsequent gestation. Only four fetuses (7.4%) developed second-degree or third-degree AVB, and one developed mild endocardial fibroelastosis.^[88]^ Based on these findings, it is recommended to administer prophylactic hydroxychloroquine in subsequent pregnancies for SLE patients with a prior history of fetal AVB. However, further evidence is required to support the prophylactic use of hydroxychloroquine in SLE patients at low risk of fetal AVB.

The optimal treatment for newly identified fetal AVB remains controversial without a standard algorithm.^[89]^ Case reports have described the use of dexamethasone, plasma exchange, IVIG, β agonists, rituximab, and immunosuppressants; however, their efficacy has not been definitively established yet. Notably, dexamethasone possesses anti-inflammatory properties due to its resistance to inactivation by 11β-hydroxysteroid dehydrogenase in the placenta, allowing favorable biological activity and potential transport to the fetus. Consequently, it is recommended by certain experts. One prospective, open-label, non-randomized study called PR Interval and Dexamethasone Evaluation (PRIDE) involved 30 pregnancies treated with dexamethasone (including 22 with third-degree AVB, 6 with second-degree AVB, and 2 with first-degree AVB) and 10 pregnancies without dexamethasone (including 9 with third-degree AVB and 1 with first-degree AVB). The analysis revealed that all cases of third-degree AVB were irreversible, resulting in fetal death or the need for permanent pacemaker implantation. For first- and second-degree AVB cases, despite dexamethasone treatment, there was still a possibility of progression to third-degree AVB, with only a few cases showing reversal.^[90]^ Based on the available limited evidence, some experts suggest oral dexamethasone treatment at a dose of 4–8 mg/day for 1–2 weeks upon identification of first-degree AVB through fetal echocardiography. If the AVB progresses to third-degree, dexamethasone should be discontinued; however, if the AVB remains stable or reverts to sinus rhythm, a maintenance dose of 4 mg/day is recommended to prevent further progression. Several retrospective studies have indicated that dexamethasone was not effective for third-degree AVB. A systematic review analyzing 8 retrospective small-sample observational studies comprising a total of 162 cases of immune-mediated fetal third-degree AVB demonstrated that prenatal dexamethasone therapy did not show significant benefits in terms of reversing the third-degree AVB (OR = 0.9, 95% CI:0.1–15.1), pacemaker implantation after birth (OR = 1.09, 95% CI:0.4–3.4), or fetal or neonatal mortality (OR = 0.5, 95% CI:0.9–2.7). However, it was found to significantly contribute to the improvement or resolution of hydrops fetalis.^[91]^

## Early Identification of Pregnancy Complications in SLE Patients, including Preterm Delivery, FGR, and PE/Eclampsia

Recommendation 8: We recommend the early identification of risk factors associated with pregnancy complications, including LN, active SLE, hypertension, positive APLs, and the use of high-dose glucocorticoids during pregnancy (strong recommendation, low-quality evidence). We suggest initiating aspirin prior to the 16^th^ week of gestation as a preventive measure to reduce the risk of eclampsia or PE in SLE patients (weak recommendation, low-quality evidence).

Pregnancy complications among SLE patients occur at a significantly higher incidence compared to the general population. These complications include premature rupture of membranes, preterm delivery, FGR, PE/eclampsia, and postpartum infection. Early identification and timely intervention are of paramount importance in preventing and managing these complications in SLE patients.

A national study analyzing over 13, 000 pregnancies in SLE patients in the US revealed a 2 to 4-fold increased rate of pregnancy complications compared to the general population.^[4]^ These complications included infection, thrombosis, thrombocytopenia, gestational diabetes mellitus,^[92]^ hypertension, PAH, and impaired renal function. Within the SLE population, 33.6% of patients required cesarean section, 20.8% experienced pre-term delivery, and 13% to 35% developed PE.

Preterm delivery is a prominent pregnancy complication among SLE patients, with a significantly higher rate compared to the non-SLE population (18% *vs*. 5%).^[93]^ The aforementioned national study conducted in the US reported an incidence of 20.8% of preterm delivery in SLE patients.^[92]^ Preterm delivery poses an important risk for neonatal complications such as infection, necrotizing enteritis, respiratory failure, intraventricular hemorrhage, neonatal hypoglycemia, neonatal jaundice, and neonatal death. Current evidence indicates that premature rupture of membranes is a major cause of preterm delivery.^[94]^ Furthermore, SLE activity and hypertension have been identified as strong predictors of pre-term delivery,^[65]^ while active LN and positive APLs are also associated factors.^[95]^

FGR is a common occurrence in SLE patients during pregnancy, with an incidence ranging from 11% to 29%, particularly in patients with LN or active disease. FGR is linked to an increased risk of perinatal mortality as well as short-and long-term neurological complications.^[96]^ The underlying mechanisms of FGR primarily involve placental insufficiency and endothelial dysfunction in spiral arteries of the placenta. Chronic exposure to glucocorticoids during pregnancy can contribute to vasoconstriction and increased arterial resistance in the placenta, further elevating the risk of FGR.^[97]^ Evidence suggests that ultrasound examination of the fetal umbilical artery is a valuable tool for early identification of FGR and predicting prognosis. However, accurate interpretation should be done by experienced sonographers, taking into consideration the gestational age as well.^[97]^

PE is characterized by new-onset hypertension (blood pressure ≥140/90 mmHg, 1 mmHg = 0.133 kPa) accompanied by proteinuria (24-hour urine protein ≥0.3 g). PE with severe features is defined by manifestations such as severe hypertension (blood pressure ≥160/100 mmHg), microangiopathic hemolytic anemia, thrombocytopenia, elevated lactate dehydrogenase, increased liver enzymes, epigastric pain, symptoms of central nervous system ischemia (*e. g*., nausea, vomiting, visual disturbance, and stroke), heavy proteinuria, or increased serum creatinine. The diagnosis of eclampsia is made when a grand mal seizure occurs. The underlying mechanisms of PE in SLE patients are yet to be fully elucidated, but several studies have suggested a potential role of angiogenic factors and complement activation. Additionally, in SLE patients, LN, thrombocytopenia, hypocomplementemia, and positive APLs are risk factors for PE, along with predisposing factors observed in the general population (*e.g*., advanced age, prior history of PE, family history of PE, multiple pregnancy, chronic hypertension, diabetes, obesity, and chronic kidney disease).^[15]^ A study conducted in Norway evaluated 180 pregnant women with SLE, revealing a significantly increased risk of PE in patients with active SLE compared to the non-SLE population (OR = 5.33, 95% CI:2.63–10.79), while stable SLE did not show a similar risk.^[35]^ Furthermore, a meta-analysis involving 32, 217 pregnant women indicated that the use of aspirin during pregnancy among high-risk women was associated with a 10% reduction in the incidence of PE.^[98]^ Therefore, for SLE patients with the aforementioned risk factors, initiating aspirin prior to the 16^th^ week of gestation is recommended to reduce the risk of PE. In certain situations (*e. g*., advanced maternal age, multiple positive APLs, positive LAC, in vitro fertilization, *etc*. ), the addition of LMWH to aspirin may be advisable. However, the dosage and duration of therapy should be individualized based on the patient’s condition.^[99]^

In SLE patients who require immunosuppressants to control the disease, immune suppression is experienced, resulting in a significantly higher risk of infection during pregnancy. A meta-analysis demonstrated a considerably higher incidence of postpartum infection in SLE patients compared to the non-SLE population (RR = 4.35, 95% CI:2.69–7.03).^[100]^ Additionally, a population-based cohort study reported that SLE patients were 1.7 times more likely to experience infection during the hospitalization period following birth (RR = 1.7, 95% CI:1.4–2.0), with a higher risk observed in patients with renal disease (RR = 3.3, 95% CI:2.3–4.7). However, no significant difference was observed in the risk of chorioamnionitis.^[101]^

## Indications for Delivery and Determination of Delivery Method

Recommendation 9: We suggest delivery when patients reach the 39^th^ week of gestation with stable conditions and mature fetuses and we suggest vaginal delivery in cases where there is no specific indication for cesarean section (weak recommendation, low-quality evidence). We recommend terminating the pregnancy as soon as possible if any of the following conditions are present: significantly active disease in the first trimester, severe SLE posing a threat to maternal safety, placenta insufficiency jeopardizing fetal wellbeing, severe gestational hypertension, neuropsychiatric SLE, cerebrovascular accidents, diffuse parenchymal lung diseases leading to respiratory failure, severe PAH, and a 24-hour urine protein level of ≥3 g (strong recommendation, very-low-quality evidence).

For patients with stable SLE, delivery is suggested at the 39^th^ week of gestation. It is recommended to discontinue aspirin use from the 36^th^ week onwards to minimize the risk of perioperative bleeding. Patients receiving LMWH should be stopped at least 12–24 h before delivery. After delivery, if no significant bleeding occurs, the original dose of LMWH should be restarted as early as possible. It is important to note that SLE itself is not an indication for cesarean section, and the mode of delivery should be determined by obstetricians based on the individual patient’s condition.

When maternal and fetal safety is at risk due to the activity of SLE, early termination of pregnancy should be considered. A significantly active SLE during the first trimester, characterized by SLE flares, active LN, cerebrovascular accidents, and other manifestations, significantly increases the incidence of adverse pregnancy outcomes.^[102, 103, 104]^ In cases where ultrasound or electronic fetal monitoring reveals placental insufficiency or when serious SLE poses a threat to maternal safety,^[105]^ preterm delivery becomes necessary.^[106]^ The presence of active SLE, particularly in individuals with LN, can result in gestational hypertension. If hypertension is poorly controlled and progresses to severe gestational hypertension, PE, eclampsia, or HELLP syndrome, prompt delivery becomes critical to preserving the lives of both the mother and the fetus. Furthermore, pulmonary or neuropsychiatric involvement can further exacerbate pregnancy outcomes.^[107,108]^ Maternal mortality rates are considerably higher in cases of diffuse parenchymal lung diseases with respiratory failure, neuropsychiatric abnormalities, and cerebrovascular accidents.^[109,110]^ Therefore, timely delivery should be considered in such circumstances. Some SLE patients may also suffer from PAH, which can worsen during pregnancy, particularly in the second and third trimesters. Additionally, new-onset PAH can develop in SLE patients during pregnancy.^[111,112]^ When the disease progresses to a moderate to severe state, maternal mortality rates are significantly increased.^[113]^ Moreover, progressively increasing urinary protein levels or a 24-hour urine protein measurement ≥3 g indicate poor control of SLE and may lead to adverse pregnancy outcomes. In such cases, timely delivery should be considered to mitigate the adverse effects on the mother.^[65,114]]^

If delivery is planned before the 34^th^ week of gestation based on the patient’s condition, the administration of fluorine-containing glucocorticoids is recommended to facilitate fetal lung maturation. This should be initiated within one week of the intended delivery time. The suggested regimen consists of 5 mg or 6 mg of intramuscular dexamethasone administered every 12 h for a total of four doses, which should be completed no later than 24 h before delivery. Alternatively, 12 mg of betamethasone can be given intramuscularly once a day for a total of two days.^[115]^

Regarding the use of glucocorticoids, for patients who are in stable condition and receiving oral prednisone at a dose of ≤5 mg per day (or an equivalent dose of other oral glucocorticoids), it is recommended to continue the original dose during induced abortion, vaginal delivery, or cesarean section. In cases where the dose of chronic oral glucocorticoids exceeds prednisone 5 mg per day (or an equivalent dose of other glucocorticoids), or if patients present symptoms of Cushing’s syndrome, additional glucocorticoids are advised perioperatively to prevent adrenal insufficiency.^[116]^ Specifically, for induced abortion or vaginal delivery, an additional 5 mg of oral prednisone or 25 mg of intravenous hydrocortisone should be administered on the day of the procedure or at the onset of labor, with the original dose resumed the following day. For cesarean section, an additional 50–75 mg of intravenous hydrocortisone should be given before or during the operation on the day of the procedure. From the next day, the dose should be changed to an additional 20 mg of intravenous hydrocortisone every 8 h, and the original oral dose should be resumed on the third postoperative day.^[117,118]]^

## Breast-feeding

Recommendation 10: We recommend breastfeeding for SLE patients who are willing and have no contraindications (strong recommendation, low-quality evidence). We suggest the use of lactation-compatible drugs to maintain disease stability, including oral glucocorticoids, hydroxychloroquine, azathioprine, and calmodulin inhibitors (weak recommendation, low-quality evidence). We recommend against the use of cyclophosphamide, mycophenolate mofetil, leflunomide, and methotrexate during breastfeeding (strong recommendation, low-quality evidence).

Breastfeeding offers various benefits to infants, providing them with high-quality nutrition while bolstering their immune system and reducing the risk of developing future diseases, such as obesity, diabetes, heart disease, and malignant tumors.^[119, 120, 121, 122]^ Postpartum SLE patients are at risk of disease flares; therefore, the use of lactation-compatible medications can help maintain their condition. Research has shown that low doses of oral glucocorticoids have no detrimental effects on infants, allowing normal breastfeeding when patients are on an oral prednisone dose of less than 20 mg per day (or an equivalent dose of other glucocorticoids). However, if the prednisone dose exceeds or is equal to 20 mg per day (or an equivalent dose of other glucocorticoids), a 4-hour interval between medication intake and breastfeeding is recommended to minimize glucocorticoid exposure in infants.^[123,124]^

Hydroxychloroquine is recommended for continuous use after delivery as it has the potential to reduce the risk of postpartum SLE flares. It exhibits low excretion into human milk and no confirmed adverse effects on infant development have been reported.^[125]^ Azathioprine can be used postpartum with close monitoring for adverse events as its metabolite, 6-mercaptopurine, is present in low concentrations in human milk.^[126,127]^ Additionally, cyclosporine A and tacrolimus have minimal concentrations in breast milk, making them eligible for postpartum use with the need for blood concentration monitoring if deemed necessary.^[128, 129, 130, 131]^

Conversely, cyclophosphamide,^[132]^ mycophenolate mofetil, leflunomide, and methotrexate ^[133]^ are contraindicated during lactation due to their potential impact on infant development and the lack of relevant safety data. Biologic drugs such as rituximab and belimumab should also be avoided due to limited safety data available for their use during lactation.

## Identification and Treatment of NLS

Recommendation 11: We recommend conducting a thorough examination of the neonate’s skin, heart, liver, blood system, and nervous system if the mother with SLE tests positive for anti-SSA antibodies and/or anti-SSB antibodies (strong recommendation, very-low-quality evidence). We recommend an immediate referral to pediatric cardiologists and consideration for the implantation of a permanent pacemaker if necessary in cases where NLS with cardiac involvement is diagnosed (strong recommendation, low-quality evidence). We recommend symptomatic and supportive treatment for NLS patients with manifestations other than cardiac involvement (strong recommendation, low-quality evidence). We suggest regular follow-up appointments for infants until 9 months to 1 year of age or until serum anti-SSA antibodies and/or anti-SSB antibodies become negative (weak recommendation, very-low-quality evidence).

NLS is a rare autoimmune disease characterized by the passive transfer of maternal autoantibodies, including anti-SSA antibodies, anti-SSB antibodies, and anti-U1 ribonucleoprotein antibodies. It is not exclusive to neonates born to SLE patients but can also affect neonates of Sjogren’s syndrome patients or asymptomatic carriers of anti-SSA antibodies and/or anti-SSB antibodies. NLS primarily presents with skin lesions, cardiac damage, hepatobiliary involvement, and hematopenia.

The incidence of NLS among SLE patients positive for anti-SSA antibodies and/or anti-SSB antibodies is approximately 5%. The key clinical manifestations of NLS are outlined below: (1) Skin, Around 40% of NLS patients exhibit skin lesions; however, only 20% of these patients are born with these manifestations. The typical presentation occurs within three months after birth, often triggered by sun exposure. These skin lesions resemble subacute cutaneous lupus and are characterized by oval or annular pink-red macules or scaling plaques. Targetoid lesions with central duskiness and discoid lesions may also be present. The lesions predominantly occur in sun-exposed areas, with the face (particularly around the eyes, giving rise to an eyeshade-like or raccoon-like appearance), perioral region, and malar and temporal areas being the most common sites. The lesions generally regress by 6–9 months of age without scarring or pigmentation.^[134,135]^ (2) Heart: Cardiac involvement in NLS extends beyond the conduction system and encompasses various manifestations, including myocardiopathy (see recommendation 7 for further details). (3) Hepatobiliary system: Approximately 10%-25% of NLS patients develop hepatobiliary manifestations such as asymptomatic elevation of transaminases, hepatomegaly, and increased γ-glutamyl transferase levels. Hepatobiliary damage can occur in isolation or in conjunction with cutaneous or cardiac involvement.^[134,135]^ (4) Hematological system: Anemia and thrombocytopenia are the typical hematological findings in NLS patients, while neutropenia and aplastic anemia are relatively less common. Hematological symptoms can be observed in around 10%-20% of NLS cases. (5) Other manifestations: Neurological impairment, characterized by macrocephaly with or without hydrocephalus, is a relatively rare occurrence in NLS.

As infants with NLS continue to mature, maternal autoantibodies gradually clear from their system, resulting in the gradual resolution of symptoms, except for cases of heart block. Data indicates that only 10% of NLS patients remain positive for anti-SSA antibodies by the age of 9 months. Cutaneous, hepatobiliary, hematological, and neurological manifestations commonly improve spontaneously within 6 to 8 months without the need for treatment, and complications are rare. However, patients with cardiac abnormalities should be promptly referred to pediatric cardiologists, particularly those with third-degree AVB who may require permanent pacemaker implantation. Sunscreen should be used for NLS patients with skin lesions to protect them from ultraviolet light exposure. In cases where hepatobiliary damage is severe or persistent, glucocorticoids can be administered at a dose of 1–2 mg·kg^-1^·d^-1,^ with a gradual reduction in dosage after the fifth day. Symptomatic patients with anemia or thrombocytopenia may require blood transfusion. For refractory anemia or thrombocytopenia, treatment options may include glucocorticoids at a dose of 1–2 mg·kg^-1^·d^-1^ for 5 days or IVIG at a dose of 1 g·kg^-1^·d^-1^ for 1–2 days. These treatment modalities can be considered on a case-by-case basis.

## Postpartum Monitoring

Recommendation 12: We suggest maintaining the original treatment strategies if SLE remains stable during delivery, conducting a follow-up visit at 4–6 weeks after delivery to assess disease activity and consider treatment adjustments if necessary, and monitoring closely until 6–12 months postpartum (weak recommendation, very-low-quality evidence). We suggest resuming prophylactic anticoagulant therapy from 12–24 h after delivery until 4–6 weeks postpartum in patients positive for APLs, and restarting the original long-term anticoagulant strategy in patients with a history of thrombosis (weak recommendation, very-low-quality evidence).

SLE patients are at a high risk of flares during the puerperium. However, there is currently no consensus on the frequency and duration of postpartum follow-ups, predictors of postpartum flares, and the impact of such flares on overall prognosis. It is important to note that maternal deaths in the puerperium are often caused by thrombotic events, underscoring the need for prophylactic treatment for thrombosis in high-risk patients.

Several prospective clinical studies have reported rates of postpartum SLE flares ranging from 0.36 to 1.80 person-year.^[21,42]^ A study published in 1996 found that pregnant SLE patients had a higher risk of flares within 8 weeks after delivery compared to age-matched non-pregnant SLE patients.^[136]^ Analysis of data from 304 patients with 398 pregnancies in the US Johns Hopkins cohort revealed a high risk of flares within 12 weeks postpartum.^[42]^ A nationwide prospective observational study in Norway, involving 145 SLE patients, assessed disease activity during pregnancy and the first year postpartum and found higher activity at 6 and 12 months after delivery compared to the third trimester and 6 weeks postpartum. Based on these findings, it is recommended to closely monitor disease activity and improve disease control between 6 and 12 months after delivery.^[137]^ If SLE patients have been receiving glucocorticoids, hydroxychloroquine, azathioprine, or calcineurin inhibitors before delivery, continuation of these medications postpartum is suggested, with the flexibility to adjust the drugs as per the patient’s condition under the guidance of physicians.

SLE patients face a heightened risk of venous thromboembolism during the puerperium period; however, there is a dearth of high-quality evidence to guide prevention strategies. Currently, management decisions primarily rely on factors such as APLs, thrombosis risk, prior history of thrombosis, and mode of delivery. For patients with APS who have received prophylactic dose heparin during pregnancy, the EULAR recommends continuing heparin at the same dosage for 6 weeks following delivery to mitigate thrombosis risk. However, there is a lack of clinical evidence supporting the continuous use of prophylactic heparin in this context.^[138]^

## Vaccinations for the Offspring of SLE Patients

Recommendation 13: We recommend following the standard vaccination schedule for neonates of SLE patients who do not have congenital immunodeficiencies or contraindications (strong recommendation, low-quality evidence). We suggest avoiding live attenuated vaccines for the offspring within 6 months after birth if SLE patients are treated with biologic drugs during the second and third trimesters or while breastfeeding (weak recommendation, low-quality evidence).

The presence of autoantibodies and the use of biologic drugs in SLE patients can potentially impact the immune system of their neonates. The question of whether neonates can receive vaccinations as per schedule to achieve active immunization is a crucial clinical concern, but the available evidence is limited to case reports. Therefore, in the absence of high-quality evidence, clinical guidelines largely stem from expert consensus.

During the third trimester, IgG antibodies can cross the placental barrier and reach the fetus through active transport. Consequently, in SLE patients receiving biologic medications, these drugs can be detected in neonatal cord blood and peripheral blood, potentially suppressing the immune system and increasing the risk of infections. Moreover, these medications may attenuate immune responses to vaccinations. The long-term impact of biologic drugs on immune system development remains uncertain. A review reported that six neonates with rituximab exposure did not experience adverse outcomes or impaired immune responses after receiving inactivated vaccinations. However, one neonate exposed to infliximab tragically died from disseminated Bacillus Calmette-Guérin infection after vaccination.^[139]^

With reference to the international guidelines on neonatal vaccinations and Chinese expert consensus on vaccinations among children with health issues,^[140]^ it is recommended that neonates of SLE patients receive inactivated vaccines according to the standard schedule, provided they have no congenital immunodeficiencies or contraindications related to allergies to vaccine components. However, if the SLE patients have received biologic medications during the second and third trimesters or while breastfeeding, it is advisable to avoid administering live attenuated vaccines to their offspring within the first 6 months after birth. This precaution is necessary to prevent the risk of disseminated infection, which includes vaccines such as the oral rotavirus vaccine, polio vaccine, measles-mumps-rubella vaccine, Bacillus Calmette-Guérin vaccine, and others.

It is important to note that maternal autoantibodies can also cross the placenta through active transport, potentially impacting the immune responses of the fetuses. Some neonates may exhibit NLS manifestations, and there is a possibility of immune response activation following vaccinations. A study reported two cases of NLS with cutaneous manifestations, which were temporarily exacerbated by vaccinations. However, the lesions resolved spontaneously without requiring specific treatment.^[141]^
